# Modeling Dendrimers Charge Interaction in Solution: Relevance in Biosystems

**DOI:** 10.1155/2014/837651

**Published:** 2014-02-27

**Authors:** Domenico Lombardo

**Affiliations:** Consiglio Nazionale per le Ricerche-Istituto per i Processi Chimico-Fisici (CNR-IPCF), Viale F. Stagno d'Alcontres 37, 98158 Messina, Italy

## Abstract

Dendrimers are highly branched macromolecules obtained by stepwise controlled, reaction sequences. The ability to be designed for specific applications makes dendrimers unprecedented components to control the structural organization of matter during the bottom-up synthesis of functional nanostructures. For their applications in the field of biotechnology the determination of dendrimer structural properties as well as the investigation of the specific interaction with guest components are needed. We show how the analysis of the scattering structure factor *S(q)*, in the framework of current models for charged systems in solution, allows for obtaining important information of the interdendrimers electrostatic interaction potential. The finding of the presented results outlines the important role of the dendrimer charge and the solvent conditions in regulating, through the modulation of the electrostatic interaction potential, great part of the main structural properties. This charge interaction has been indicated by many studies as a crucial factor for a wide range of structural processes involving their biomedical application. Due to their easily controllable properties dendrimers can be considered at the crossroad between traditional colloids, associating polymers, and biological systems and represent then an interesting new technological approach and a suitable model system of molecular organization in biochemistry and related fields.

## 1. Introduction

A new class of regularly branched macromolecules, called dendrimers, has recently received a stimulating and growing interest in the field of nanotechnology. These synthetic macromolecules consist of relatively short chains with specific functional groups at the ends. Their structure, which is obtained by means of suitable iterative stepwise reactions sequences, is developed through successive generations and gives rise to monodisperse, highly branched tree-like “dendritic” morphology [1, 2]. Due to their peculiar arrangement and their easily controllable properties, dendrimers can be considered at the crossroad between traditional colloids, associating polymers, and biosystems [3, 4]. The potential of these new polymeric compounds can be utilized for the development of new prototypes and new materials which will contribute to clear up the understanding of basic processes in a wide range of research fields including material science, photonics, macromolecular biology, biophysics, and medicine [5, 6]. It is clear then how a detailed and complete knowledge of the structural features of starburst dendrimer, together with their relevant parameters, can be useful for the engineering of new technologies as well as clearing up the understanding of some basic processes which are common in different fields of science, such as structural organisation, self-assembly, transport processes, and molecular recognition.

## 2. Structure and Properties of Dendrimers

**Figure 1:**
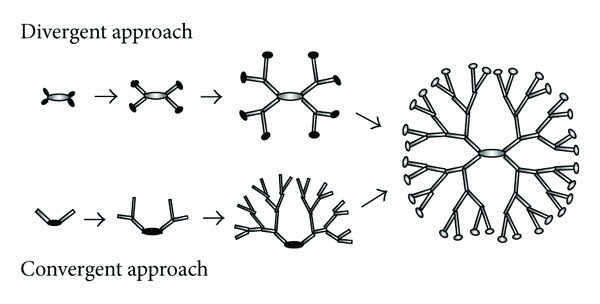
Sketch of the two synthesis protocols for the construction of dendritic macromolecules. Black regions highlight the functional sites.

Dendrimers structures consist of *branching units* covalently attached to a *central core,* which are organized in concentric layers (named generations) and a number of external *surface functional groups*. The iterative stepwise reaction sequence used for dendrimer synthesis permits a marked control over critical molecular design parameters (such as size, shape, and internal/surface chemistry) which results in highly ramified structures and high monodispersity (typical polydispersity index is *M*_W_/*M*_N_ < 1.01). As depicted in Figure 1, two major strategies (viz. the *divergent* and *convergent *methods) have been developed for dendrimer synthesis. In the divergent approach, first introduced by Tomalia [1], starting from a central reactive core, successive generations are grown at each step of the synthesis, while the new peripheral molecules are activated for successive reaction with further monomers. In each generation step, the molar mass of the dendrimer is nearly doubled. In the convergent method, first introduced by Hawker and Fréchet [3], a multifunctional core reacts with several dendrons, while the anchoring of the branches to the core furnishes the final hyperbranched product. While the divergent approach is suitable for the production of large quantities of dendrimers, the convergent method has the advantages of simplicity of the purification of the final reaction product (with minimal synthesis defects) and the precise placement of functional moieties at the dendrimers periphery.

On the basis of these two synthetic strategies, a relevant number of compositionally different dendrimer families have been synthesized while a wide range of differentiated chemical surface modifications have been reported in literature. Although different types of starburst dendrimers have been synthesized in the last decades, polyamidoamine (PAMAM) dendrimers represent the first dendrimers family to be commercialised and actually the most investigated typology [4]. PAMAM dendrimers are synthesized by means of the divergent method starting from an ethylenediamine or amine core and are commercially available up to the generation of G = 10 and diameters of about 15 nm.

## 3. Structural Properties of Dendrimers in Solution

Structural properties of dendrimers in solution, fundamental information intimately connected with their functions, have been extensively studied by means of both experimental [7–11] as well as theoretical methods [12–17]. Among the experimental methods, the scattering techniques such as small angle scattering of X-rays (SAXS) and neutrons (SANS) are probably the most important and widely utilized experimental approach employed for the structural investigation of biosystems in solution [18–20].

**Figure 2:**
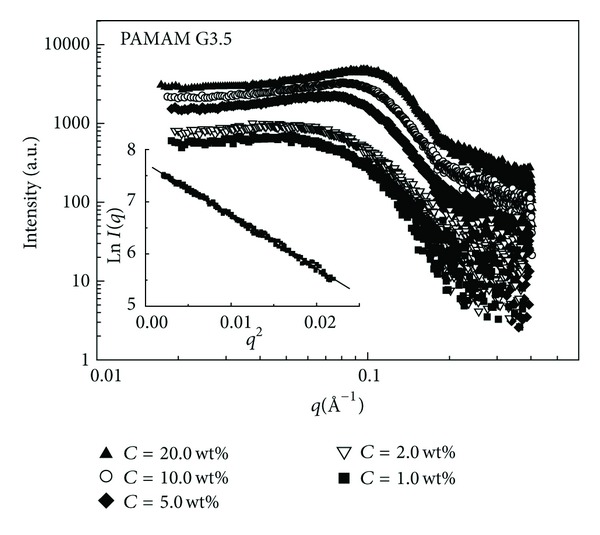
SAXS intensity profiles of methanol solution of generation G3.5 (sodium carboxylate terminated) PAMAM dendrimers at different concentrations at *T* = 23°C.

In Figure 2 the SAXS intensity profiles of methanol solution of sodium carboxylate terminated; generation G3.5 PAMAM dendrimers are reported at different concentrations at the constant temperature of *T* = 23°C. Assuming the dendrimer solution as a monodisperse system of particles, the small angle scattering intensity *I*(*q*) can be expressed as a product of the form factor *P*(*q*), which contains information on the shape and dimension of the scattering particles, and the structure factor *S*(*q*) describing the interparticle interaction [18]:

(1) $$\begin{array}{c} I\left(q\right)=N{\left(\Delta \rho \right)}^{2}P\left(q\right)S\left(q\right), \end{array}$$

where *N* is the number density of the particles and Δ *ρ* = (*ρ* − *ρ*_0_) is the so-called “contrast” (i.e., the difference between the scattering length density of the particle *ρ* and that of the solvent *ρ*_0_). In the dilute region the interparticle interaction can be neglected (i.e., *S*(*q*) ~ 1), so that the analysis of scattering intensity *I*(*q*) can furnish direct information of morphological features of the scattering particles. Assuming our dendrimers as monodisperse uniform sphere of radius *R*, the corresponding form factor can be written as *P*(*q*) = [3*J*_1_(*qR*)/(*qR*)]^2^ [18] (where *J*_1_(*x*) = [sin(*x*) − *x* cos⁡(*x*)]/*x* ^2^ is the first-order spherical Bessel function). Information about the dendrimer radius of gyration *R*_g_ can also be obtained from the slope of ln⁡*I*(*q*) versus *q* ^2^ in the so called Guinier region (i.e., for *qR*_g_ ≪ 1), where the particle form factor can be expressed as *P*(*q*)≅*P*(0) · exp⁡(−*q* ^2^ *R*_g_^2^/3). An example of the Guinier SAXS analysis is presented in the inset of Figure 2.

A first theoretical attempt to analyse the structure of dendrimers was made by de Gennes and Hervet [12]. Through a self-consistent field analysis they modeled a dendrimer of flexible trifunctional monomers and very long spacers in an athermal solvent and found that the density profile inside the dendrimer is minimal at the core and grow monotonically at the outer regions (hollow core conformation) while the size of the dendrimer was found to scale with the number *N* of monomer according to the law *R* ~ *N* ^1/5^, with an effective fractal dimension of *D*_f_ = 5. Numerical simulations results, on the other hand, show rather open structures in which the free ends of the starburst are distributed throughout the molecule, not exclusively on the surface as de Gennes and Hervet had assumed. The finding of different numerical simulations studies [13–17] predicted, in fact, a monotonically decreasing density profile from the central core, with the free ends of the starburst distributed throughout the molecule. Most of the results seem to be oriented toward a compact space-filling internal structural configuration (i.e., *D*_f_ = 3) especially for high generation (G > 4) dendrimers. A first simulation of dendrimers based on a kinetic growth model based on self-avoiding walks was performed by Lescanec and Muthukumar [13]. They found a density profile that decreases monotonically outward from the center of the molecule, with significant chain folding. The dendrimer size was found to scale with the number of monomers and the spacer length as *R* ~ *N* ^*ν*^ *n* ^*β*^, with *ν* = 0.22 and *β* = 0.50. A molecular dynamics study performed in 1996 by Murat and Grest [14] also gives evidence of a density distribution which is maximal at the center and decays to the edge of the dendrimer with a sensitive free ends chain folding and a dendrimers fractal dimension of about *D*_f_ = 3. In the same year also Boris and Rubinstein [15] by means of a self-consistent mean field model found that density profiles decrease monotonically from the core of the dendrimer to the surface.

A recent small angle X-ray scattering (SAXS) and Quasi Elastic Light Scattering (QELS) [11] study of PAMAM dendrimers of different generations (2 ≤ G ≤ 9) in methanol solution investigated the scaling law relating the dendrimer molecular mass *M*_W_ and the measured radii *R* according to the law *M* ~ *R* ^*D*_f_^ (where *D*_f_ is the fractal dimension, a scaling exponent describing the use of space in the growth of the molecule). In that case a well-defined crossover in the scaling behavior *M* ~ *R* ^*D*_f_^, passing from low to high generations dendrimers, was evidenced. More specifically on increasing generation G, the internal dendrimer structure evolves from an open self-similar density distribution (at low generations 2 < G < 5) characterized by a fractal dimension of *D*_f_ = 2.4 to a spherical homogeneous and compact one (at the higher generations 6 < G < 9) with a fractal dimension of *D*_f_ = 4.8.

**Figure 3:**
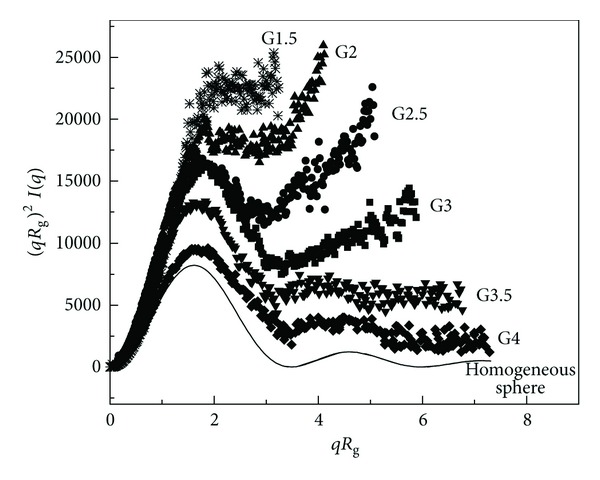
Kratky representation of the form factor corresponding to different generation of PAMAM dendrimers in methanol solution at the concentration of *C* = 1  wt.%.

In Figure 3 a modified Kratky representation of the PAMAM dendrimers form factor *P*(*q*) (obtained by plotting the (*qR*_g_)2*I*(*q*) versus *qR*_g_) allow us to remove the length scale dependence and accentuate the contributions due to the internal density configuration. Compared with the form factor for hard spheres (uniform solid particles), it is clear that higher generation (G ≥ 3) assumes a more homogeneous and compact structure configuration in solution.

In conclusion, with the increasing number of branch units with larger generations, the core region becomes increasingly shielded off from the surroundings, while with the generated dense surface configuration the formation of compartments in the dendrimers interior regions is favoured. In this case the specific microenvironments may be exploited to carry low-molecular substances such as (hydrophobic) drugs.

## 4. Modeling Dendrimers Charge Interaction

The structure of dendrimers in solution can be influenced by many factors, such as the generation, spacer length, surface modification, ionic strength, pH, and temperature. On the other hand there are the charge effects and electrostatic self-assembly processes that seem to play the main role in the effective synthetic strategy to generate highly stable, dendrimer-based nanotechnologies. In this respect it is of fundamental importance to obtain details on the interdendrimer charge interaction in different solution environment.

The first computational [21] and experimental [22] studies dealing with the modeling of charged dendrimers in solution have been carried out in the early 1998. By using Monte Carlo (MC) simulations Welch and Muthukumar [21] described charged PAMAM dendrimers in aqueous solutions in terms of the Debye-Huckel potential to approximate the repulsive Coulomb interactions. In that case the dendrimer radial density profile undergoes a conformational transition from a compact to an extended profile under a decrease in the salt concentration.

**Figure 4:**
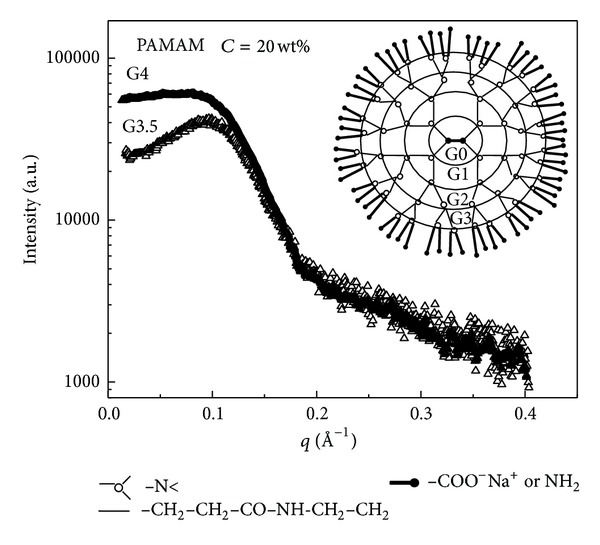
SAXS spectra of amine terminated (G4) and sodium carboxylate terminated (G3.5) PAMAM dendrimers in methanol solution at the concentration of *C* = 20 wt.%. The presence of a peak in the SAXS spectra (more pronounced for the G3.5 system) gives indication that a long range structural order between dendrimers of electrostatic origin is present in solution.

In Figure 4 the presence of a peak in the SAXS spectra of G4 and G3.5 PAMAM dendrimers in methanol solution gives indication that a long range structural order (due to the charged dendrimers surface) can be explained in terms of the electrostatic repulsive interaction produced between dendrimers. From the experimental point of view the framework for calculating the electrostatic potential of a charged system involves the equations of classical electrostatics and is treated in the framework of DLVO model [23–25]. In order to obtain information about the interdendrimer interaction potential the charged dendrimers can be approximated as impenetrable spheres of radius *R* whose charge Ze is distributed on the surface. Those spheres are immersed in the uniform neutralising background of the solvent molecules which participates with its dielectric constant and which produces also a screening effect in the system. According to this model the repulsive potential between two identical spherical objects (macroions) of diameter *σ* = 2*R* placed at a distance *r* (centre-to-centre distance) are given by [23, 24]:

(2) $$\begin{array}{c} U\left(r\right)=\frac{Z_{0}e^{2}}{4\pi \varepsilon {\left(1+\kappa \sigma \right)}^{2}}\frac{e^{-\kappa (r-\sigma )}}{r}. \end{array}$$

Here *κ* is the Debye-Huckel inverse screening length which is determined, at a given temperature *T*, by the ionic strength *I* of the solvent (in mol/L), according to the following relation:

(3) $$\begin{array}{c} \kappa =\sqrt{\frac{8\pi e^{2}N_{a}I}{\varepsilon K_{B}T\times 10^{3}}}, \end{array}$$

where *e* is the unit of electron charge, *K*_*B*_ is the Botzmann constant, *N*_*a*_ is the Avogadro number. We also assume a hard sphere type repulsive component for the potential to represent the close contact interdendrimer interaction.

**Figure 5:**
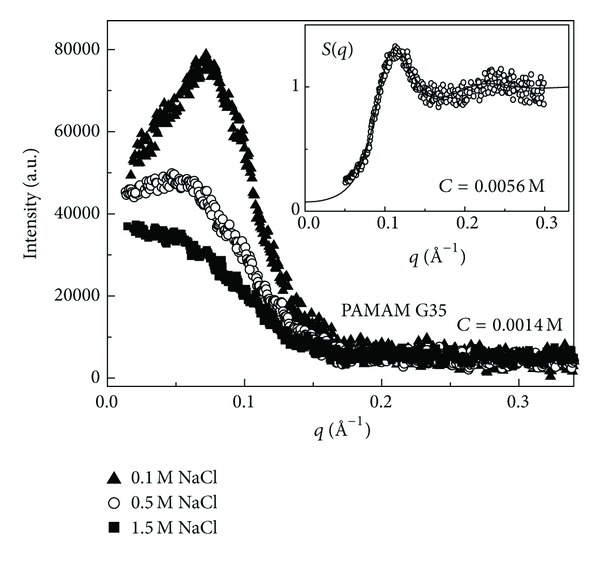
Evolution of the SAXS intensity profiles upon addition of different amount of NaCl electrolytes in a water solution of G3.5 PAMAM dendrimers at the concentration of *C* = 0.0014 M. Modeling of the interdendrimers structure factor *S*(*q*) at the higher concentration of *C* = 0.0056 M (inset).

In Figure 5 the effect of the screening of the interdendrimers electrostatic interaction potential is presented in water solution of sodium carboxylate terminated G3.5 PAMAM dendrimers. SAXS data evidence how addition of different amount of NaCl electrolytes results in a screening effect for the interaction potential, as confirmed by the disappearing of the characteristic peak in the SAXS spectra. According to the DLVO model [23] the equilibrium structural properties of a macroion solution are computed by numerical methods starting from the knowledge of some structural parameters, such as the particles concentration (in mol/L), the effective charge Ze, and the particle diameter *σ*. According to this model the observed interdendrimer structure factors *S*(*q*), obtained by SAXS experiments, can be calculated by means of the solution of the Ornstein-Zernike equation [24] and the use of suitable closure relations [25]. More specifically the structure factor *S*(*q*) for a system of interacting particles can be written as

(4) $$\begin{array}{c} S\left(q\right)=1+\int_{0}^{\infty }4\pi^{2}\rho_{C}\left[g\left(r\right)-1\right]\frac{\sin\left(qr\right)}{\left(qr\right)}dr. \end{array}$$

This provides a way to relate the structure factor *S*(*q*) with the radial pair correlation function *g*(*r*) (i.e., the probability that two particles stay at distance *r* in the system) and then, by means of a suitable closure relation, to the interparticle potential *U*(*r*) [24, 25]. The result of this procedure, by using the HNC closure relation [25], is presented in the inset of Figure 5. From the figure it is clear how an effective dendrimer charge of *Z* = 24*e* (were *e* is the electron charge) can reproduce quite satisfactorily the finding of the experimentally determined structure factor *S*(*q*).

Recent SANS scattering studies of the interdendrimers interactions in aqueous (D_2_O) solution of (positively charged) amine terminated PAMAM dendrimers indicated the structural effects caused by the protonation of the amino end-groups upon the addition of acid [26, 27]. The influence of this additional electrostatic interaction opens the prospect of using this charge-stimulated conformational transition to facilitate possible applications in dendrimer-based hosts/guest systems.

## 5. Biotechonlogical Applications of Dendrimers

One of the most frequent applications of dendrimers consists in conjugating chemical species to the dendrimer surface that can function as detecting agents, affinity ligands, targeting components, or imaging agents [1, 2]. Dendrimers have also been tested in preclinical studies as contrast agents (e.g., for magnetic resonance applications) [2, 4]. Moreover, the combination of high surface area and high solubility makes dendrimers useful as nanoscale catalysts [4, 5]. Dendrimer applications as light harvesting indicate that the harvested light energy can be transformed into chemical energy for reactions, electric current, or converting the energy into monochromic light [6].

Recently the development of a dendrimer-based host system obtained by a synthesis process involving zeolite growth on PAMAM dendrimers as template has been presented [28, 29]. The use of the PAMAM dendrimer templates seems to be an interesting possibility, in substitution of more traditional templates for the development of porous biomaterials. The structural similarities between the substrate-binding sites of enzymes and the zeolites cavities may lead to the development of mesoporous particles capable of mimicking the enzyme functions, where mesoporous barriers may promote selective reactions and incorporation of key features of selected enzymes. This study stimulated the successful investigation on the formation processes of hybrid nanoparticles and the development of novel approaches to prepare organic, inorganic, and biological nanomaterials [30, 31].

In solution environments the wide range of tunable properties makes dendrimers a versatile tool as host trough the inclusion of guest molecules in their interior voids or attaching guest molecules in the dendrimers surface [32].

**Figure 6:**
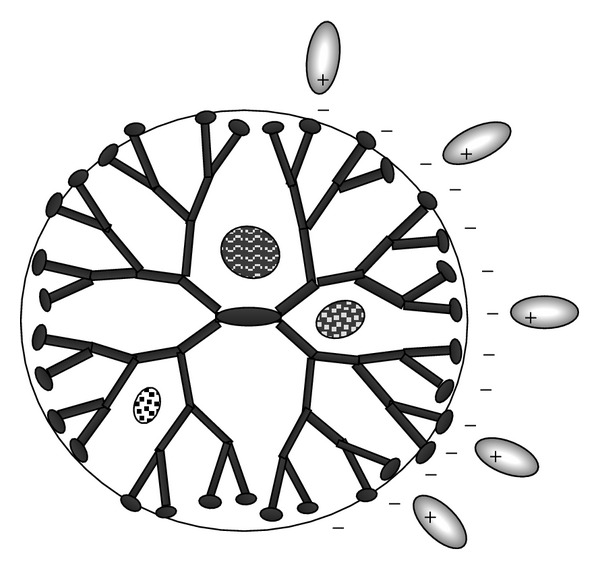
Possible mechanism of interaction between dendrimers and drugs molecules.

As presented in Figure 6, there are two main approaches that have received much attention for the development of dendrimers-based application in nanotechnology:

- encapsulation of guest molecules in the internal cavities of the dendrimer;
- external electrostatic (or covalent) attachment of components onto dendrimer surfaces.

Attachment by electrostatic binding at the surface and interior encapsulation (by hydrophobic interactions and hydrogen-bond interactions) was proposed as the major interaction mechanisms between dendrimers and guest molecules in drug delivery applications. Despite the number of recent investigations of dendrimer-based host-guest systems [33], much of the properties about the formation of dendrimers-based complex still remain unknown.

Noncovalent (or covalent) attachment of drugs to dendrimers was reported to significantly affect the dissolution rate, the aqueous solubility, the stability and other physicochemical properties of the drugs in physiological conditions [33, 34]. For example, it has been shown that dendrimers are effective for the treatment of chronic inflammatory disorders [35]. More specifically, the amino- (G4-NH_2_) and hydroxyl-terminated (G4-OH) PAMAM dendrimers revealed a much stronger anti-inflammatory effects with respect to the carboxy-terminated (G4-CO_2_H) dendrimers.

### 5.1. Dendrimer-Polyelectrolytes Interaction

Particularly interesting for biomedical applications is the electrostatic interactions of polyelectrolytes systems and charged dendritic nanoparticles, as the corresponding complex formation represents a basic phenomenon in many biological systems [5]. Fluorescence spectroscopy investigations indicate the important role of charge interaction in dendrimers binding with double-stranded DNA [36], human serum albumin [37], and bovine serum albumin [38]. More specifically, when DNA is mixed with PAMAM dendrimers, it undergoes a transition from a semiflexible coil to a more compact conformation due to the electrostatic interaction present between the cationic PAMAM dendrimers and the anionic DNA polyelectrolyte.

A recent study indicated that human serum albumin (HSA) binding constants (Kb) of Pamam dendrimers depend on size and terminal group chemistry and suggested several mechanisms of interactions between PAMAM dendrimers and HSA proteins [37] including:

- electrostatic interactions between charged dendrimer terminal groups and protein residues;
- hydrogen bonding between dendrimer internal groups (e.g., amide moiety where the carbonyl O acts as donor and the amide H as acceptor) and protein amino acid residues;
- hydrophobic interactions between HSA groups and the nonpolar dendrimer;
- specific interactions between binding sites (aliphatic acid) of proteins and dendrimer carboxylic groups.

With the aim to describe the phase-transition-like behavior for the interaction of polyelectrolytes with oppositely charged particles, different experimental studies have been performed on micelles- [39], dendrimers- [40], and protein-polyelectrolyte [41] systems that revealed the existence of a critical conditions for complexation. Recently, complex formation between generation 3 carboxyl-terminated dendrimers and cationic polyelectrolytes (of varying linear charge density) was studied as a function of ionic strength, by turbidimetric titration and dynamic light scattering [42]. In that case at low (or moderate) ionic strength (*I*) the critical surface charge density *σ*_c_ (at the point of incipient complex formation) has been shown to be roughly proportional to *κ*/*ξ*, where *κ* is the Debye-Hückel parameter and *ξ* is the linear charge density of the polyelectrolyte.

### 5.2. Dendrimer-Surfactant Interaction and Translocation in Biomembranes

Another relevant aspect of charge-mediated self-assembly processes involving dendrimers regards the study of the formation of dendrimer-surfactant (lipids) complexes. It is well known that in surfactant systems the thermodynamic incompatibility between the different blocks causes a microphase separation that gives rise to a variety of spatial organization of morphologies [43–46]. The combination of supramolecular interactions, together with the ability to control both the length scale and the structural morphologies, makes surfactant systems particularly attractive components for biotechnological applications [47–49]. This self-assembly may have important implications for the understanding of translocation mechanism of dendrimers and biomacromolecules in living cells. In this respect, several model systems that mimic the structure of biomembranes were developed during the last decades [50–53]. Depending on dendrimer chemical composition, size and surface charge different mechanisms can be identified to rule out the main interactions between dendrimers and lipid bilayers, including *adsorption on membrane, hole formation, *and* vesicles disruption* [54–57]. The different mechanisms of interaction strongly depend on the force balance between charged dendrimers and the zwitterionic lipids (that have a net dipolar charge) and on the hydrophobic interaction between the arms of the dendrimers and the lipids hydrocarbon chains. For example, adsorption of PAMAM dendrimers on phosphatidylcholine bilayers investigated by molecular simulations indicated the crucial role of the electrostatics interaction as the main driving force that regulate adsorption on membranes. Hole formation occurs for larger generation dendrimers mainly, and it is proposed to be mediated by the formation of lipid coated-dendrimer complexes (named dendrisomes). Finally high generation dendrimer may act as a bridge between two vesicles while the packing stresses, due to the local inversion of curvature in the bilayer, will lead to enhanced lipid mixing between the neighboring vesicles with a consequent vesicle disruption (and leakage of internal aqueous content) [54]. A recent study indicates that amine terminated (positively charged) polypropylenimine dendrimers interacting with the plasma membrane of cultured cells induced hole formation and allow the transport of dendrimer across the plasma membrane, while amide/PEG modified dendrimer exhibits no membrane disruption or intracellular accumulation [55]. Concerning PAMAM interactions with small unilamellar lipid vesicles, in some cases they do not induce content leakage from egg phosphatidylcholine (PC) vesicles [56], indicating that the interactions are barely electrostatic and dendrimers mainly adsorb weakly onto the vesicle surface. A recent dynamic light scattering (DLS), cryo-TEM, and small angle X-ray scattering (SAXS) investigation [57] indicated that the electrostatic interaction favorites the linking between POPC lipid vesicles at physiologically relevant conditions in presence of generation G = 6 Pamam dendrimers, while no hole or dendrosome formation has been evidenced. Moreover, at high dendrimer/lipid ratio dendrimer addition leads to collapse of the vesicles with formation of a gel phase.

**Figure 7:**
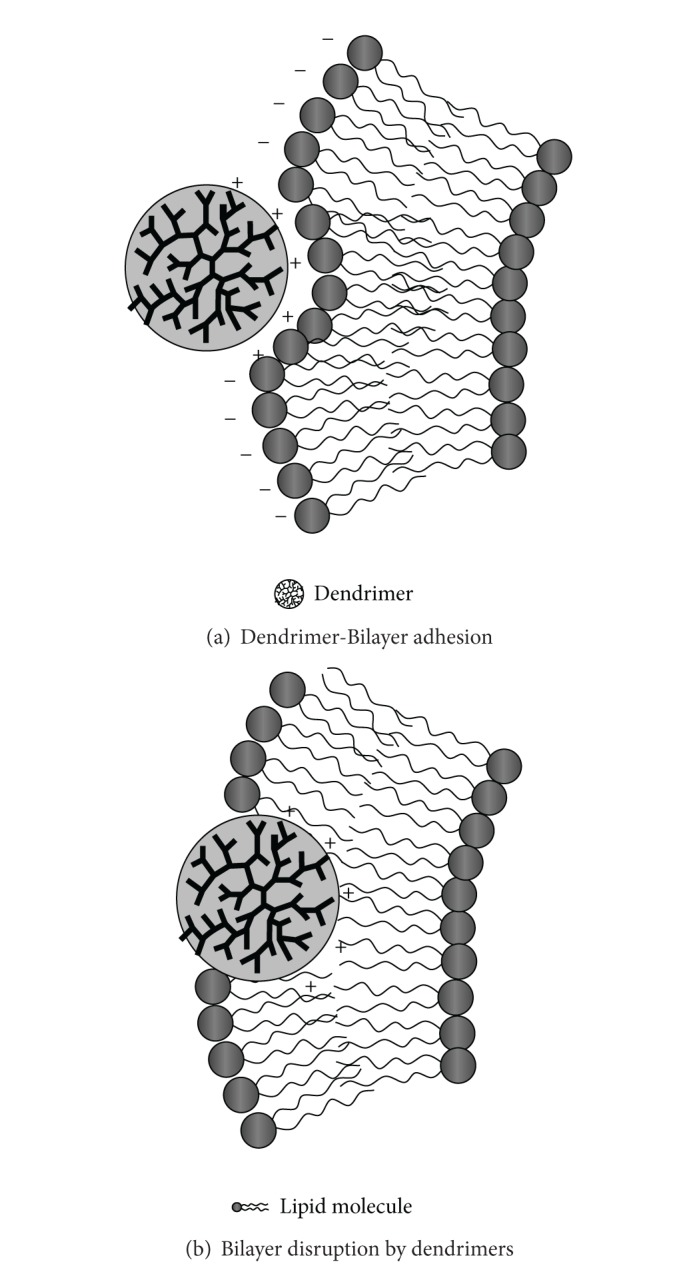
Example of dendrimer-lipid interaction in biomembranes.

Recently a comprehensive study of the interactions in dendrimer-lipid complex identified some structural transitions dependent strongly on the stoichiometry and structure of the main components such as dendrimer generation and termination and phospholipid headgroup (see Figure 7) [58].

Finally dendrimer-DNA complexes (called dendriplexes) revealed such efficient systems able to mediate (nonspecific) *in vitro* transfer of genetic material into different cell lines. Recently Kukowska-Latallo et al. [59] indicated that generation G3-G10 PAMAM dendrimers form complexes with DNA under most physiologic conditions that are able to efficiently mediate (nonspecific) *in vitro* transfer of genetic material into different cell lines. Under physiological conditions, dendriplexes maintain a positive net charge and bind to negatively charged surface molecules on the cell membranes. Dendrimers-DNA complexes are taken up into cells by nonspecific endocytosis and are then degraded by lysosomes. The targeting genes are then released and enter the nucleus to play a role in gene therapy. Transfection efficiency, mediated by PAMAM dendrimers, appears to be dependent on dendrimer generation and on the charge ratio of the complexes.

### 5.3. Drug Targeting in Bioactive Dendrimers for Clinical Applications

A new emerging field of clinical application concerns the combination of dendrimers and bioactive ligands. Dendrimer conjugates containing saccharides or peptides may exhibit therapeutic application for the development of antimicrobial, antiprion, and antiviral agents. Moreover, they offer additional advantages for their versatile capabilities to enhance solubility, stability and absorption of various types of therapeutics. Together with the covalent bonding, the electrostatic interaction plays a crucial role in the development of methods to load the dendrimer scaffolds with therapeutics. In this case, charged therapeutics form complexes by ionic binding with dendrimers containing counter-charged groups. This approach has been used for nucleic acid-based therapeutics and other charged therapeutic [60]. In those cases, the controlled release of noncovalently bound drugs may be driven by concentration gradients or by pH-triggered conformational change of the dendrimer [61]. The positive charge of either amines terminated or guanidine-modified Pamam dendrimers has been shown to prevent prion folding and induce prion unfolding with decreased cytotoxicity due to dendrimer glycosylation [62], while antimicrobial application of modified dendrimers with positive peptides evidenced a strong toxicity and selectivity action for bacteria. Moreover, glycodendrimers have demonstrated antiviral properties by presenting saccharides to either directly bind to viruses or to saturate cell surface receptors [63, 64]; both methods inhibit virus-cell interaction. Drug-dendrimer complex for targeted delivery can also exploit the host-guest hydrophobic interactions in cyclodextrin-conjugated dendrimers through the inclusion of the drug molecule into the cavity of the cyclodextrin. This approach facilitated the solubilization of a wide range of hydrophobic small molecules involved in antidepressant, anti-inflammatory, anticancer, and antimicrobial applications [65].

### 5.4. Dendrimers-Based Technologies for Biorecognition and Detection

**Figure 8:**
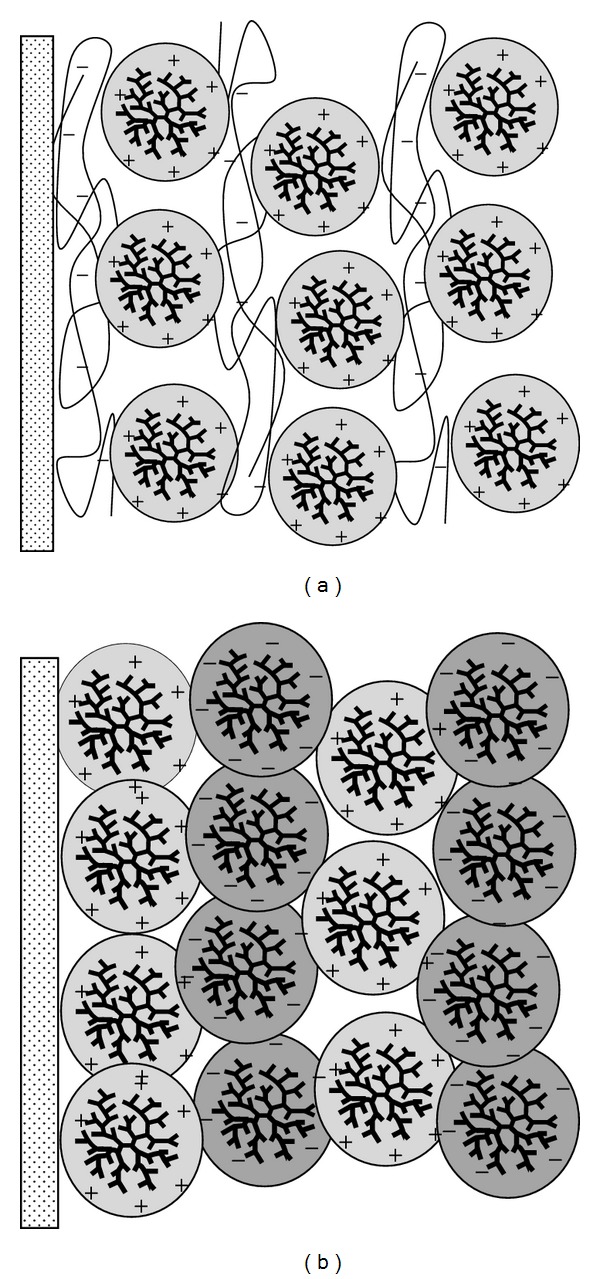
Example of polymer/dendrimer (a) and dendrimer/dendrimer (b) layer-by-layer (LbL) assemblies.

Another interesting application of dendrimers is the biodetection systems (biosensors) which are fabricated using electrodes or optical transducers coupled with molecular recognition elements (i.e., enzymes and antibodies). The immobilization on a support is the most important step-process to construct enzyme-based detection technologies. In this respect a widely used configuration in medicine and biotechnology is the dendrimer-based Layer-by-Layer (LbL) assemblies. LbL films are constructed using positively charged dendrimers combined with polyanions (or negatively charged dendrimers and polycations) as depicted in Figure 8(a). Alternatively, oppositely charged dendrimers may be used for self-assembly of dendrimer LbL films without the intervention of polymers (Figure 8(b)).

Very often also hydrogen or covalent bonding or biological affinity is exploited as complementary driving force for the formation of LbL films. In order to improve the performance of biosensors [66, 67] deposition of LbL multilayer films composed of dendrimers and proteins has been widely employed, followed by the encapsulation (or covalent binding) of metal nanoparticles or electron transfer mediators. For example dendrimers-based LbL FET sensors with tetrasulfonated phthalocyanine (TsPc) sensitive to pH [68], humidity [69], and glucose [70] have been recently realized. In this case the high performance of the FET sensors has been ascribed to the porous structure of the TsPc/dendrimer LbL films permeable to H+ and to glucose.

## 6. Conclusions

The construction of supramolecular nanostructures based on the versatile use of building blocks has been drawing increasing attention for its high efficiency to create functional nanomaterials starting from a variety of organic, inorganic, and biological basic components. In this respect, the highly controllable features such as their size, shape, and surface functionality make dendrimers a versatile component in a wide range of biochemical applications in the field of nanotechnology. Conjugation of chemical species into the dendrimer surface gives rise to the development of new prototypes that can function as detecting, targeting, or imaging agents, while drug delivery applications indicated an efficient use of dendrimers for (*in vitro*) transfer of genetic material into cells. A quantitative analysis of the physical interactions between dendrimers and inclusion components is a crucial step for the development of novel technology. In this respect, the small angle scattering techniques represent powerful approaches to study the structural properties of dendrimers in solution environment. In this respect, the modeling of the interdendrimer interaction provides substantial insight into the fundamental mechanisms of dendrimer-agents interaction in solution. Notably, solution conditions (including solvent pH, counterion distribution, and ionic strength) have been shown to play a key role in the control of the charge interaction and can be exploited in the rational design of dendrimer properties for suitable applications in nanotechnology. The recent literature indicates that encouraging results have been obtained for the rational design and the developments of novel prototypes able to achieve additional degree of control over the fundamental properties of the dendrimer-based technology, while the information obtained could indicate the preferred strategy for cost-effective synthesis protocols, and thus opening the way for the successful commercialization of this emerging technology.
